# Deletion of Trace Amine-Associated Receptor 1 Attenuates Behavioral Responses to Caffeine

**DOI:** 10.3389/fphar.2018.00035

**Published:** 2018-02-02

**Authors:** Michael D. Schwartz, Jeremiah B. Palmerston, Diana L. Lee, Marius C. Hoener, Thomas S. Kilduff

**Affiliations:** ^1^Center for Neuroscience, Biosciences Division, SRI International, Menlo Park, CA, United States; ^2^Neuroscience, Ophthalmology and Rare Diseases Discovery and Translational Area, Pharma Research and Early Development, Roche Innovation Center Basel, F. Hoffmann-La Roche, Ltd., Basel, Switzerland

**Keywords:** TAAR1, sleep, arousal, psychostimulants, caffeine, dopamine, adenosine, addiction

## Abstract

Trace amines (TAs), endogenous amino acid metabolites that are structurally similar to the biogenic amines, are endogenous ligands for trace amine-associated receptor 1 (TAAR1), a GPCR that modulates dopaminergic, serotonergic, and glutamatergic activity. Selective TAAR1 full and partial agonists exhibit similar pro-cognitive, antidepressant- and antipsychotic-like properties in rodents and non-human primates, suggesting TAAR1 as a novel target for the treatment of neurological and psychiatric disorders. We previously reported that TAAR1 partial agonists are wake-promoting in rats and mice, and that TAAR1 knockout (KO) and overexpressing mice exhibit altered sleep-wake and EEG spectral composition. Here, we report that locomotor and EEG spectral responses to the psychostimulants modafinil and caffeine are attenuated in TAAR1 KO mice. TAAR1 KO mice and WT littermates were instrumented for EEG and EMG recording and implanted with telemetry transmitters for monitoring locomotor activity (LMA) and core body temperature (T_b_). Following recovery, mice were administered modafinil (25, 50, 100 mg/kg), caffeine (2.5, 10, 20 mg/kg) or vehicle p.o. at ZT6 in balanced order. In WT mice, both modafinil and caffeine dose-dependently increased LMA for up to 6 h following dosing, whereas only the highest dose of each drug increased LMA in KO mice, and did so for less time after dosing. This effect was particularly pronounced following caffeine, such that total LMA response was significantly attenuated in KO mice compared to WT at all doses of caffeine and did not differ from Vehicle treatment. T_b_ increased comparably in both genotypes in a dose-dependent manner. TAAR1 deletion was associated with reduced wake consolidation following both drugs, but total time in wakefulness did not differ between KO and WT mice. Furthermore, gamma band EEG activity following both modafinil and caffeine treatment was attenuated in TAAR1 KO compared to WT mice. Our results show that TAAR1 is a critical component of the behavioral and cortical arousal associated with two widely used psychostimulants with very different mechanisms of action. Together with our previous findings, these data suggest that TAAR1 is a previously unrecognized component of an endogenous wake-modulating system.

## Introduction

Considerable progress has been made in identification of both wake- and sleep-promoting systems over the past two decades. Although the monoaminergic systems were originally thought to facilitate sleep (Jouvet, 1969), subsequent research established that activation of the noradrenergic, serotoninergic and histaminergic systems promote wakefulness (Saper et al., 2010; Scammell et al., 2017). Similarly, midbrain dopaminergic systems have been shown to promote wakefulness (Lu et al., 2006; Eban-Rothschild et al., 2016; Cho et al., 2017), in contrast to early studies suggesting lack of state-dependent activity (Miller et al., 1983). The discovery of the hypocretin/orexin (Hcrt) system (de Lecea et al., 1998; Sakurai et al., 1998) added yet another wake-promoting system, with the additional feature that the Hcrt provided excitatory input to the aforementioned monoaminergic as well as the cholinergic systems in the basal forebrain and midbrain (Schwartz and Kilduff, 2015). More recently, two wake-promoting GABAergic neuronal populations have been found in the lateral hypothalamus (Herrera et al., 2016; Venner et al., 2016). These discoveries have demonstrated that the maintenance of wakefulness involves highly redundant systems that extend well beyond the classical ascending reticular activating system (Moruzzi and Magoun, 1949).

Trace amines (TAs) are endogenous amino acid metabolites that are structurally similar to the biogenic amines (Grandy, 2007). TAs such as beta-phenylethylamine (β-PEA), tyramine, octopamine and tryptamine are endogenous ligands for trace amine-associated receptor 1 (TAAR1), a GPCR that negatively modulates monoaminergic and glutamatergic activity (Borowsky et al., 2001; Bunzow et al., 2001). TAAR1 is expressed in cortical, midbrain and limbic forebrain regions important for behavioral arousal and motivation (Bunzow et al., 2001; Xie et al., 2007; Lindemann et al., 2008). Selective TAAR1 agonists exhibit pro-cognitive, antidepressant and antipsychotic properties (Revel et al., 2012b, 2013), and have been shown to ameliorate addictive behaviors in a number of paradigms (Lynch et al., 2013; Pei et al., 2014; Thorn et al., 2014; Cotter et al., 2015; Liu et al., 2017). Previously, we reported that TAAR1 partial agonists promote wakefulness in WT rats (Revel et al., 2012b, 2013) and mice (Schwartz et al., 2017), and that TAAR1 knockout (KO) and overexpressing mice exhibit altered sleep-wake and EEG spectral composition (Schwartz et al., 2017). Both full and partial TAAR1 agonists also suppress REM sleep and alleviate cataplexy in mouse models of narcolepsy (Black et al., 2017a), suggesting utility for TAAR1-directed compounds in treating sleep disorders. Based on these findings, we proposed that TAAR1 is a previously unrecognized component of an endogenous wake-promoting system (Schwartz et al., 2017). Here, we tested whether TAAR1 deletion altered wake-promoting responses to two common and widely used psychostimulants, caffeine (Caf) and modafinil (Mod).

Among exogenous wake-promoting substances, Caf is undoubtedly the most widely consumed psychostimulant worldwide. Caf antagonizes receptors for adenosine, an endogenous ATP metabolite that accumulates during prolonged wakefulness and is proposed to mediate the homeostatic drive to sleep (Strecker et al., 2000; Halassa et al., 2009; Clasadonte et al., 2014). Although Caf was initially thought to counteract sleepiness by antagonizing the adenosine A1 receptor (Virus et al., 1990; Benington et al., 1995), more recent studies have implicated the adenosine A2a receptor (Huang et al., 2005; Lazarus et al., 2011), particularly in the nucleus accumbens (Lazarus et al., 2011). Among prescription medications, Mod was the first compound marketed as a wake-promoting therapeutic to counter the excessive daytime sleepiness of narcoleptic patients. Although the mechanism of its action remains controversial, Mod binds at low affinity to the dopamine transporter (DAT) (Mignot et al., 1994) and the wake-promoting actions of Mod are abolished in DAT knockout mice (Wisor et al., 2001) although other mechanisms of action for Mod have also been suggested (Touret et al., 1994; Garcia-Rill et al., 2007; Duchene et al., 2016).

Irrespective of the receptor site(s) at which Caf and Mod bind, relatively little attention has been paid to the downstream neural circuitry through which these compounds effect their wake-promoting actions and the interaction with the currently known wake-promoting systems (Deurveilher et al., 2006; Lazarus et al., 2011; Zhang et al., 2013). Accordingly, in the present study we asked whether TAAR1- a negative modulator of dopaminergic tone in the ventral tegmental area, striatum and the accumbens (Lindemann et al., 2008; Revel et al., 2011; Leo et al., 2014; Espinoza et al., 2015a) – is involved in the neural circuitry mediating wake promotion by Caf and Mod.

## Materials and Methods

### Animals

Adult, 3-month old male homozygous *Taar1* KO mice (*n* = 9) and their WT littermates (*n* = 11) maintained on a pure C57BL/6 background were bred at SRI International. The generation and breeding of these mice has been previously described (Lindemann et al., 2008; Revel et al., 2012a). All KO and WT mice in the present study were bred from 11 het/het pairs of *Taar1* transgenics. All mice were singly housed in polyethylene cages (280 mm × 175 mm × 130 mm) with extended vertical Plexiglas sides that permitted the animal to be tethered for EEG/EMG recordings. Cages were kept inside ventilated, light-tight sound-attenuated chambers in a 12:12 h light-dark cycle; within each chamber, light was provided by white LEDs connected to a programmable timer, yielding 40 lx at cage level during the light phase; lights came on at 08:00 Pacific Standard Time). Temperature and humidity were maintained at 22 ± 2°C and 50 ± 25% respectively. Food and water were available *ad libitum* for the duration of the study. Every effort was made to minimize animal discomfort throughout the experimental protocols. All studies were conducted in accordance with the *Guide for the Care and Use of Laboratory Animals* and were approved by the Institutional Animal Care and Use Committee at SRI International.

### Surgical Procedures

Mice were instrumented for tethered EEG and EMG recording and implanted with intraperitoneal telemetry transmitters for monitoring of locomotor activity (LMA) and core body temperature (T_b_) as described previously (Fisher et al., 2013, 2016). Under isoflurane anesthesia and aseptic conditions, the intraperitoneal cavity was accessed via a midline incision, the peritoneum was irrigated with physiological saline, a sterile telemetry device (G2 E-Mitter; Phillips Respironics, Bend, OR, United States) was sutured to the inner abdominal muscle, and the incisions were then closed with absorbable sutures. Next, a dorsal midline incision was made on top of the head, the temporalis muscle was retracted, and the skull was cleaned with a 3% hydrogen peroxide solution. A prefabricated EEG/EMG headmount (8201-C; Pinnacle Technologies, Lawrence, KS, United States) was affixed to the skull with four stainless steel screws that acted as EEG electrodes. Screws were positioned approximately 1.5 mm lateral to the sagittal suture, with frontal screws located 2.0 mm anterior of bregma and posterior screws -4.0 mm posterior to bregma. The threads of the screws were coated with conductive silver epoxy (SEC1233; Resinlab, Germantown, WI, United States) and conductivity was tested with a multimeter. Two stainless steel braided EMG wires attached to the headmount were inserted into the trapezius muscle and sutured in place. The implant was secured to the cranium using dental acrylic (Lang Dental Manufacturing Co) and the incision was sutured. Mice were administered analgesics (buprenorphine, 0.05–0.1 mg/kg and ketoprofen, 2–5 mg/kg) and 0.9% physiological saline s.c. for 1–2 days post-surgery, maintained with thermal support and given nutrient gel and/or soft chow. Sutures were removed after 12–14 days recovery.

### EEG/EMG, LMA and T_b_ Recording and Analysis

EEG/EMG data were continuously recorded using iox2 (v2.8.0.11; EMKA Technologies, France) on a PC and analyzed as described previously (Fisher et al., 2013, 2016; Schwartz et al., 2017). Flexible cables were used to connect mouse headmounts to swivel commutators (Pinnacle Technologies) mounted above the cage’s center, allowing unrestricted movement across the entire cage. Mice were habituated to cables for at least 4 days prior to the start of data collection. Electrophysiological signals were amplified with Grass Model 15 amplifiers; EEG signals were high- and low-pass filtered at 0.3 and 300 Hz, respectively, and EMG signals were high- and low-pass filtered at 3 Hz and 6 KHz, respectively. Amplified electrophysiological signals were sampled at 500 Hz. Simultaneously with collection of EEG and EMG data, LMA and T_b_ were recorded from the implanted E-Mitters at 1-min intervals via inductive telemetry using ER-4000 receiver bases (MiniMitter/Philips Respironics, Bend, OR, United States) located beneath the home cages and connected to a PC running Vitalview (v5.0; MiniMitter/Philips Respironics).

EEG and EMG data were visually scored offline in 10 s epochs for behavioral state (Wake, REM, NREM) by expert scorers blind to genotype and drug treatment groups. Epochs that contained mixed states or recording artifacts were included in the behavioral state analysis but excluded from subsequent spectral analysis. Individual state data were quantified as time spent in each state per 1 or 6 h. Latency to NREM and REM onset for each animal was calculated from the time of drug injection (ZT6). Bouts were defined as a minimum of two consecutive epochs of any state. EEG power spectra were calculated for each state and drug condition using Fourier-transformed EEG signals (0–100 Hz, 0.1 Hz bins). For each mouse, EEG spectral values for each drug condition were normalized to the mean spectral power during the vehicle treatment for that mouse to determine EEG spectral changes relative to the vehicle. LMA and T_b_ data were subsequently analyzed using ClockLab (Actimetrics; Evanston, IL, United States).

### Drugs

Modafinil (Mod) was purchased from Waterstone Technology (Carmel, IN, United States). On the day of dosing, Mod was suspended and sonicated for 2 h in 1.25% hydroxypropyl methyl cellulose (HPMC) with 0.1% dioctyl sodium sulfosuccinate (DOSS; 2.24 mM) in sterile water (hereafter referred to as ‘Veh’) at final dosing concentrations. Caffeine (Caf) was purchased from Sigma and was dissolved in Veh at 2 mg/mL stock and diluted to final dosing concentrations on day of dosing. All mice received p.o. Mod (25, 50, 100 mg/kg), Caf (2.5, 10, 20 mg/kg) or Veh via oral gavage in the mid-light phase [Zeitgeber Time (ZT) 6, where ZT0 = lights-on and ZT12 = lights-off; thus, all drugs were given 6 h after lights-on, or 14:00 local time] with each mouse receiving all 7 drug conditions, each condition balanced across treatment days and genotypes with at least 3 days between each drug treatment. ZT6 was chosen as a time of day for treatment for two reasons: first, at this time spontaneous wakefulness is at a relatively low level, facilitating detection of any increases in wakefulness from these basal levels; second, dosing in the middle of the light phase avoids the possible confounding effects of elevated homeostatic sleep drive that exist near the start of the light (resting) phase or the elevated circadian drive for wakefulness that occurs near the start of the dark (active) phase in nocturnal rodents (Trachsel et al., 1986; Borbely and Achermann, 2000). Oral dosing was chosen to correspond to the typical route of administration for both Mod and Caf in humans. Mice were acclimated to oral dosing with gavage needles by dosing with vehicle once per day for 3 days, with acclimation ending at least 3 days prior to data collection.

### Statistical Analyses

Drug efficacy was evaluated within genotypes using two-way within-subjects ANOVAs comparing drug treatment (Veh, Mod 25/50/100 mg/kg, Caf 2.5/10/20 mg/kg) and time (h), and between genotypes using two-way mixed ANOVAs comparing genotype and drug treatment. Positive ANOVA results were followed by *post hoc* Bonferroni *t*-tests or planned comparison *F* tests where appropriate. Bout architecture results were analyzed within genotype using two-way within-subjects ANOVAs comparing drug treatment and bout duration; positive results were followed by planned comparisons of drug treatment vs. Veh within each bout duration category. 60-Hz noise was filtered out of all spectral analyses by removing all values between 59.8 and 60.2 Hz. Total power for each of 6 frequency bands (delta, 0.5–4 Hz; theta, 4.5–9 Hz; alpha, 9–12 Hz; beta, 12–30 Hz; low gamma, 30–59.8 Hz; high gamma, 60.2–100 Hz) was compared via two-way mixed ANOVAs comparing genotype and drug treatment, followed by *post hoc* planned comparison *F*-tests.

## Results

### Locomotor Activity

**FIGURE 1:**
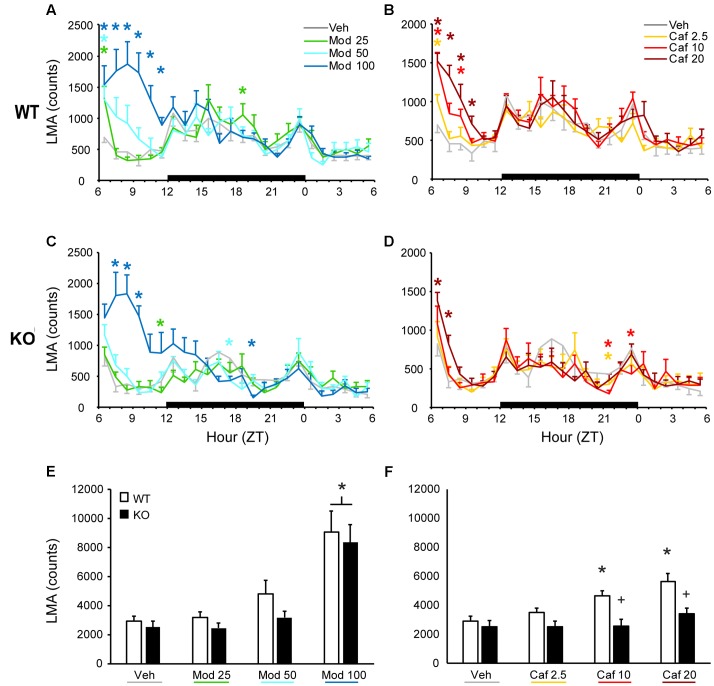
In WT mice, both Mod **(A)** and Caf **(B)** increased LMA for up to 6 h following dosing at ZT6 whereas, in TAAR1 KO mice, only the highest dose of each drug increased LMA and did so for less time **(C,D)**. In WT mice, Mod (**E**; 100 mg/kg) and Caf (**F**; 10–20 mg/kg) increased total LMA summed over 6 h post-dosing relative to vehicle whereas, in KO mice, only Mod (100 mg/kg) increased total LMA relative to vehicle. Furthermore, total LMA following Caf (10–20 mg/kg) was significantly attenuated in TAAR1 KO mice compared to WT. ^∗^*p* < 0.05 vs. Veh. +*p* < 0.05 vs. WT.

In WT mice, all doses of Mod (**Figure 1A**) and Caf (**Figure 1B**) increased LMA compared to Veh for at least 1 h and up to 6 h following dosing [*F*_(138,1380)_ = 3.25, *p* < 0.001], whereas only the highest dose of each drug increased LMA in TAAR1 KO mice and did so for less time [*F*_(138,1104)_ = 3.72, *p* < 0.001; **Figures 1C,D**). To directly compare the effects of genotype on drug response, we analyzed cumulative LMA over the 6 h following dosing (ZT7-12; **Figures 1E,F**). A mixed-model ANOVA comparing genotype and response to all seven drug treatments yielded significant effects of genotype [*F*_(1,18)_ = 5.83, *p* = 0.027] and drug treatment [*F*_(6,108)_ = 22.72, *p* < 0.001] without interaction. However, separate analyses of Mod and Caf responses compared to Veh revealed that Mod (100 mg/kg) increased cumulative LMA independent of genotype [*F*_(13,54)_ = 28.33, *p* ≤ 0.001; **Figure 1E**), whereas Caf significantly increased cumulative LMA in WT, but not KO, mice at 10 and 20 mg/kg compared to Veh [*F*_(3,54)_ = 2.86, *p* = 0.045; **Figure 1F**].

### Body Temperature

**FIGURE 2:**
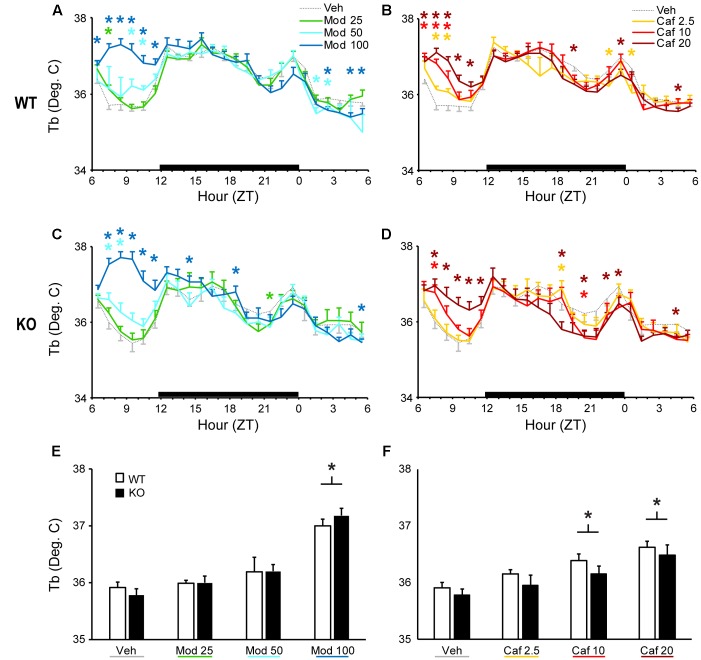
Both Mod **(A,C)** and Caf **(B,D)** dose-dependently increased hourly T_b_ for up to 6 h following dosing in both WT and TAAR1 KO mice. **(E,F)** Mod (100 mg/kg) and Caf (10, 20 mg/kg) increased average T_b_ over the 6 h following dosing but the average T_b_ increases over 6 h post-dosing did not differ between KO and WT mice. ^∗^*p* < 0.05 vs. Veh.

Both Mod and Caf dose-dependently increased T_b_ compared to Veh for up to 6 h following dosing in WT [*F*_(138,1380)_ = 2.90, *p* < 0.001; **Figures 2A,B**] and KO mice [*F*_(138,1104)_ = 3.40, *p* < 0.001; **Figures 2C,D**). Mod (100 mg/kg) and Caf (10, 20 mg/kg) both increased average T_b_ over the 6 h following dosing [*F*_(6,108)_ = 25.8, *p* < 0.001] but, in contrast to LMA, there was no influence of genotype (**Figures 2E,F**).

### Sleep/Wake States

**FIGURE 3:**
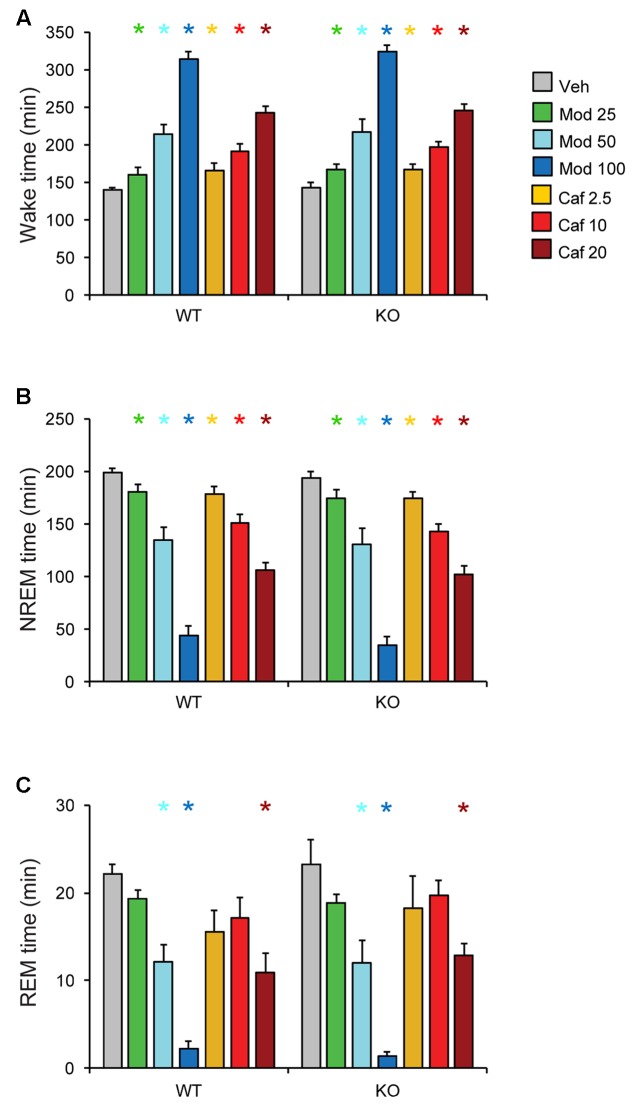
Both Mod and Caf dose-dependently increased wakefulness **(A)** and decreased total NREM **(B)** and REM **(C)** sleep time for 6 h post-dosing. There were no effects of genotype on total sleep time or hourly time courses for each state (not shown). ^∗^*p* < 0.05 vs. Veh.

**FIGURE 4:**
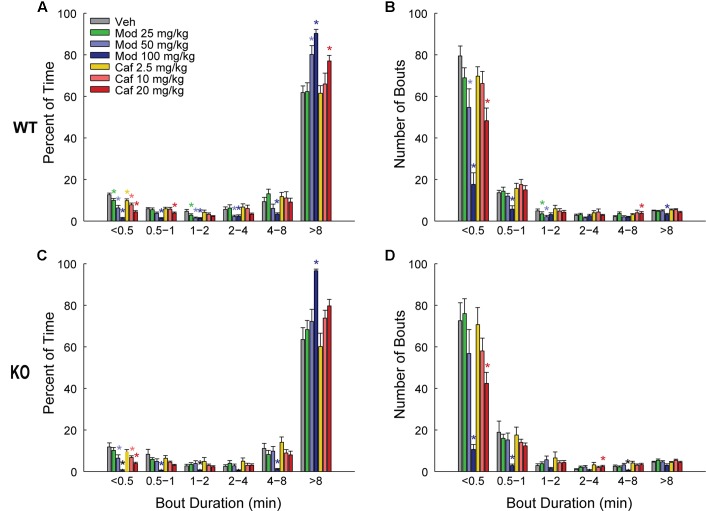
In WT mice **(A,B)**, Mod (50 and 100 mg/kg) consolidated wakefulness by increasing the percentage of Wake time spent in long (>8 min) wake bouts **(A)** and the number of 4–8 min long bouts **(B)**, at the expense of shorter wake bouts **(A,B)** but, in KO mice, only Mod 100 mg/kg exhibited comparable effects **(C,D)**. Caf (20 mg/kg) similarly consolidated wakefulness in WT mice whereas, in KO mice, Caf failed to increase the proportion of wake bouts longer than 4 min **(C,D)**. ^∗^*p* < 0.05 vs. Veh.

Both Mod and Caf dose-dependently increased total wakefulness [*F*_(6,108)_ = 90.84, *p* < 0.001] and decreased total NREM [*F*_(6,108)_ = 97.55, *p* < 0.001] and REM sleep time [*F*_(6,108)_ = 21.94, *p* < 0.001] for 6 h post-dosing (**Figures 3A–C**). There were no effects of genotype on total sleep time (**Figure 3**), hourly sleep/wake time or NREM/REM latency (not shown). Mod (50 and 100 mg/kg) consolidated wakefulness in WT mice by increasing the percentage of Wake time spent in long (>8 min) wake bouts and the number of 4–8 min wake bouts, while decreasing the proportion of shorter wake bouts [*F*_(30,300)_ = 9.00, *p* < 0.001; **Figures 4A,B**]. By contrast, only Mod 100 mg/kg comparably consolidated wakefulness in KO mice [*F*_(30,240)_ = 6.74, *p* < 0.001; **Figures 4C,D**]. Similarly, Caf (20 mg/kg) consolidated wakefulness in WT mice by increasing the percentage of Wake time in wake bouts > 8 min long (**Figure 4A**) and the number of 4–8 min wake bouts (**Figure 4B**) whereas, in KO mice, Caf failed to increase the proportion of wake bouts longer than 4 min (**Figures 4C,D**). Thus, both drugs exhibited comparable wake-promoting efficacy in WT and KO mice in terms of total wake time, but TAAR1 deletion was associated with reduced consolidation of wakefulness following both Mod and Caf.

### EEG Power Spectra

**FIGURE 5:**
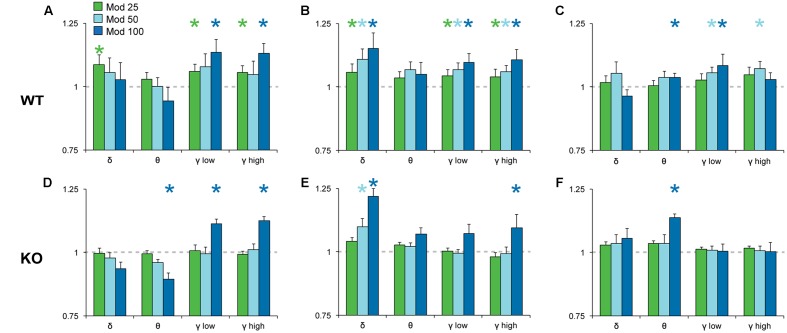
Normalized EEG power spectra in Wake **(A,D)**, NREM **(B,E)** and REM sleep **(C,F)** following Mod. In WT mice **(A–C)**, Mod increased NREM delta power (0.5–4 Hz) and gamma power in all states (low, 30–60 Hz; high, 60–100 Hz) at all doses but, in KO mice **(D–F)**, only 100 mg/kg Mod exhibited comparable effects. Mod 100 mg/kg also increased REM theta power (4–8 Hz) in both WT and KO mice **(C,F)**. Spectral power for all drug conditions are normalized to those for each individual’s Veh recording, which is shown as a dashed line. ^∗^*p* < 0.05 vs. Veh.

**FIGURE 6:**
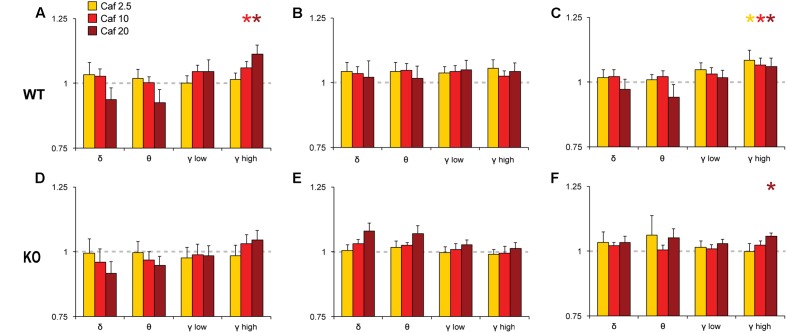
Normalized EEG power spectra in Wake **(A,D)**, NREM **(B,E)** and REM sleep **(C,F)** following Caf. In WT mice, Caf (2.5–20 mg/kg) increased gamma band activity in Wake and REM sleep **(A,C)**. In KO mice, Caf 20 mg/kg increased REM gamma power **(F)**. There were no other significant effects. Spectral power for all drug conditions are normalized to those for each individual’s Veh recording, which is shown as a dashed line. ^∗^*p* < 0.05 vs. Veh.

To analyze the effects of Mod and Caf on EEG spectral composition, we binned spectral power within each power band (see section “Materials and Methods”) for the 6 h following dosing (ZT7–ZT12) and normalized each drug treatment condition to Veh. Significant effects of drug treatment were observed for the delta [NREM, *F*_(6,108)_ = 8.74, *p* < 0.001], theta [Wake, *F*_(6,108)_ = 3.16, *p* = 0.007; REM, *F*_(6,108)_ = 2.59, *p* = 0.022], low gamma [Wake, *F*_(6,108)_ = 4.93, *p* < 0.001; NREM, *F*_(6,108)_ = 4.15, *p* < 0.001] and high gamma bands [Wake, *F*_(6,108)_ = 6.77, *p* < 0.001; NREM, *F*_(6,108)_ = 4.61, *p* < 0.001]. In addition, non-significant trends were observed for waking delta power [*F*_(6,108)_ = 2.13, *p* = 0.056] and REM high gamma power [*F*_(6,108)_ = 2.16, *p* = 0.052]. There were no effects of drug treatment on alpha or beta power in any state (not shown). While the omnibus ANOVA analyses did not reveal significant influences of genotype or genotype × drug treatment interactions, there was a widespread trend toward attenuated drug response in KO mice for the delta, theta and gamma bands (**Figures 5**, **6**). Accordingly, spectral data were further analyzed via planned pairwise comparisons of each drug vs. Veh for delta, theta and low and high gamma power within each state and each genotype (**Figures 5**, **6**). Presentation of Mod (**Figure 5**) and Caf conditions (**Figure 6**) were separated for clarity.

Pairwise comparisons showed that Mod increased NREM delta and gamma power compared to Veh at all doses in WT mice (**Figure 5B**); these effects were greatly attenuated in KO mice (**Figure 5E**). A similar pattern was observed for waking and REM spectra, with increased gamma power at multiple doses of Mod in WT mice, while only the 100 mg/kg dose exhibited comparable effects in KO mice. Mod 100 mg/kg increased REM theta power (4–8 Hz) in both WT and KO mice (**Figures 5C,F**) and suppressed waking theta power in KO mice (**Figure 5D**). Caf increased high gamma power compared to Veh in wake (10, 20 mg/kg; **Figure 6A**) and REM sleep (all doses; **Figure 6C**) in WT mice, whereas this effect was only seen in REM sleep at the highest dose in KO mice (**Figure 6F**).

## Discussion

Previously, we reported that TAAR1 partial agonists promote wakefulness in rodents (Revel et al., 2012b, 2013; Schwartz et al., 2017) and reduces cataplexy in two mouse models of narcolepsy (Black et al., 2017a), suggesting promise for treatment of this sleep disorder. Based on these findings, we tested potential mechanisms for TAAR1’s involvement in state control by comparing the response to Mod and Caf, two well-characterized psychostimulants in TAAR1-null mice and their WT littermates. In contrast to the pronounced hypersensitivity of TAAR1 KO mice to amphetamines (Wolinsky et al., 2007; Lindemann et al., 2008), we observed comparable increases in total wake time in KO and WT mice. However, KO mice exhibited mild reductions in consolidated wakefulness following both Mod and Caf compared to WT littermates, blunted EEG spectral responses to both drugs, and a strong attenuation of the motor-activating response to Caf. Constitutive TAAR1 deletion thus attenuates behavioral and EEG spectral responses to dopaminergic (Mod) and adenosinergic (Caf) stimulants, but does not grossly impair the promotion of wakefulness by these drugs.

### *Taar1* Deletion Attenuates Modafinil-Induced Locomotor Activity without Affecting Wakefulness Amounts

Elevating dopamine (DA) transmission powerfully modulates wakefulness, as indicated by the dopaminergic mechanisms of action of many potent psychostimulants, the wake-promoting roles for brainstem DA neurons (Lu et al., 2006; Eban-Rothschild et al., 2016; Cho et al., 2017), and the profound hyperarousal associated with DAT-null mutation (Wisor et al., 2001; Kume et al., 2005; Dzirasa et al., 2006). TAAR1 inhibits firing in ventral tegmental DA and dorsal raphe serotonin neurons and exhibits tonic or constitutive activity *ex vivo* (Lindemann et al., 2008; Bradaia et al., 2009; Revel et al., 2011) and *in vivo* (De Gregorio et al., 2016), which has led to the hypothesis that TAAR1-mediated inhibition negatively regulates monoaminergic tone. Consistent with this idea, TAAR1-null mice exhibit elevated basal firing rates in brainstem monoaminergic neurons, and exaggerated hyperactivity and striatal DA release following amphetamine administration (Wolinsky et al., 2007; Lindemann et al., 2008). However, constitutive TAAR1 deletion had no effect on overall promotion of wakefulness and only mild modulatory effects on the wake-consolidating efficacy of Mod. Mod promotes wakefulness via DAT inhibition (Mignot et al., 1994; Wisor et al., 2001; Wisor, 2013) and consequent elevation of extracellular DA (Wisor, 2013) and downstream DA-dependent α1 adrenergic activation (Stone et al., 2002; Wisor and Eriksson, 2005); however, wake promotion by Mod is abolished in DAT-null mice (Wisor et al., 2001), supporting a primary and essential role for that DAT. Mod and cocaine — DAT-inhibiting stimulants that do not bind TAAR1 — induce comparable levels of wakefulness and motor activation, respectively, in TAAR1 KO and WT mice (present study and Revel et al., 2011). TAAR1 is therefore not necessary for Mod-induced wakefulness, consistent with reports that TAAR1 deletion does not substantively alter DAT function *in vivo* (Leo et al., 2014).

By contrast, amphetamines and their derivatives directly activate TAAR1 (Bunzow et al., 2001), in addition to profoundly augmenting DA transmission via the DAT (Jones et al., 1998). The potentiation of amphetamine-induced LMA in TAAR1 KO mice may therefore reflect this distinct dual action on TAAR1 and the DAT. Many dopaminergic stimulants, including amphetamines, induce significant rebound hypersomnolence while others, including Mod, promote wakefulness without a corresponding sleep rebound (Edgar and Seidel, 1997; Gruner et al., 2009). This capacity for wake promotion without rebound hypersomnolence is highly desirable from a therapeutic perspective, and explains in part why Mod is so widely used to treat pathological sleepiness (e.g., in narcolepsy, Black et al., 2017b). In this light, future studies should address whether interactions with TAAR1 plays a role in rebound hypersomnolence following amphetamine administration, perhaps via its actions on non-dopaminergic systems.

### *Taar1* Deletion Attenuates Caffeine-Induced Locomotor Activity

In contrast, motor activation was significantly attenuated following Caf doses up to 20 mg/kg, while wake promotion was comparable to that of WT littermates. TAAR1 is not known to interact directly with ADO receptors, but ADO receptor activation attenuates dopaminergic activation in the spinal cord (Acevedo et al., 2016; Elnozahi et al., 2016), striatum (Ross and Venton, 2015) and nucleus accumbens (NAc) (Quarta et al., 2004), protects against DA neurotoxicity (Fathalla et al., 2016; Xu et al., 2016), and attenuates cocaine-induced locomotor and D2 receptor sensitization (Hobson et al., 2012). This ADO receptor-mediated inhibition of DA signaling is proposed to underlie the motor-activating effects of the ADO receptor antagonist Caf (Fredholm et al., 1999; Salmi et al., 2005; Ferre, 2010). TAAR1 complexes with and augments the expression, composition and function of D2 receptors (Espinoza et al., 2011; Harmeier et al., 2015). TAAR1 deletion attenuates haloperidol-induced catalepsy and striatal Fos expression (Espinoza et al., 2011), as well as quinpirole-induced locomotor inhibition (Espinoza et al., 2015a) compared to WT mice. These actions are thought to reflect attenuation and hypersensitivity of presynaptic and postsynaptic D2 receptors, respectively (Leo et al., 2014; Espinoza et al., 2015a). It is therefore possible that, in the absence of TAAR1, outputs like motor activity are less sensitive to indirect A2a-mediated D2 disinhibition following Caf and to a lesser extent Mod, leading to the unusual combination of wake promotion (via direct Caf-mediated ADO receptor antagonism) without concurrent motor activation (caused by dysfunctional D2 signaling in KOs). These effects may be mediated by TAAR1 in the basal ganglia and the NAc in particular, where ADO A2a deletion was reported to abolish wake promotion by Caf (Lazarus et al., 2011).

### *Taar1* Deletion Reduces Modafinil and Caffeine-Induced EEG Gamma Band Activity

Gamma band EEG activity is elevated in wakefulness, and changes in gamma power are related to cortical function and cognition (Sohal et al., 2009; Cho et al., 2015). NMDA receptor antagonists induce hyperactivity, aberrant cortical gamma oscillations (Pinault, 2008; Ehrlichman et al., 2009; Hakami et al., 2009) and sleep disruption (Ishida et al., 2009). TAAR1 agonists block the motor-activating and cognition-impairing effects of NMDA hypofunction (Revel et al., 2012b, 2013), while TAAR1 deletion impairs NMDA-dependent signaling in cortex and is associated with impulsive behavior (Espinoza et al., 2015b). Previously, we reported that TAAR1 deletion increases, and overexpression decreases, EEG theta and gamma power across sleep-wake state (Schwartz et al., 2017), while TAAR1 partial and full agonists acutely decrease EEG oscillations in the alpha, beta and gamma bands (Schwartz et al., 2017; Schwartz, unpublished). Here, Mod- and Caf-induced EEG gamma band activity was attenuated in KO mice. Together, these observations indicate that endogenous TAAR1 critically regulates a key neurophysiological correlate of cortical function and cognitive processing. Gamma oscillations arise from interactions between cortical pyramidal neurons and fast-spiking parvalbumin-positive interneurons (Sohal et al., 2009). The cell type in which TAAR1 is expressed in the rodent cortex is unknown but, if not on the parvalbumin neurons themselves, could be elsewhere in the microcircuit that underlies gamma oscillations.

### Perspective

TAAR1 could regulate arousal via actions on dopaminergic (Lu et al., 2006; Eban-Rothschild et al., 2016; Cho et al., 2017), serotonergic (Muraki et al., 2004; Popa et al., 2005; Buchanan and Richerson, 2010; Zant et al., 2011) or glutamatergic populations (Fuller et al., 2011; Schone et al., 2012; Kaur et al., 2013; Schone et al., 2014), all of which have been shown to modulate sleep/wake state. However, Mod- and Caf-induced wake promotion was only mildly perturbed in TAAR1-null mice, despite the documented dysregulation of monoaminergic and glutamatergic signaling in these animals (Wolinsky et al., 2007; Lindemann et al., 2008; Revel et al., 2011), suggesting that these sleep- and wake-regulatory neurotransmitter systems appear to compensate for a constitutive lack or overabundance of TAAR1. In this light, further probing of the acute interactions between TAAR1 and wake-related monoaminergic and glutamatergic circuits using selective TAAR1-directed compounds would be helpful. Alternatively, the circuits underlying TAAR1-mediated arousal may be separate from the dopaminergic (Mignot et al., 1994; Wisor et al., 2001) and adenosinergic (Rainnie et al., 1994; Huang et al., 2005) circuits mediating wake promotion by Mod and Caf, respectively. TAAR1 is enriched in mesolimbic DA nuclei including the VTA, amygdala and nucleus accumbens, but is also expressed in the cortex, hypothalamus, dorsal raphe nuclei and nucleus of the solitary tract (Bunzow et al., 2001; Lindemann et al., 2008; Espinoza et al., 2015b; Ferragud et al., 2017). Serotonergic activity has been shown to promote wakefulness and suppress REM sleep via the 5HT_1a_, 5HT_2a_, 5HT_6_ and 5HT_7_ receptors (Boutrel et al., 2002; Popa et al., 2005; Morairty et al., 2008; Monti and Jantos, 2014). Of these, TAAR1 activation is reported to modulate 5HT_1a_ signaling (Revel et al., 2011); it is currently unknown whether TAAR1 modulates other 5HT receptors as well. Future studies should assess the roles of these systems and brain regions, particularly outside the dopaminergic systems, in regulating arousal states.

## Author Contributions

MS and TK designed the study and wrote the paper. MS, JP, and DL performed the experiments and data analyses. MH contributed critical materials. MS, MH, and TK performed the critical reading of the manuscript.

## Conflict of Interest Statement

In the last 12 months, TK has received research funding from Jazz Pharmaceuticals, Inc., and Teva Pharmaceuticals United States. MH is an employee of F. Hoffmann-La Roche. The other authors declare that the research was conducted in the absence of any commercial or financial relationships that could be construed as a potential conflict of interest. The handling Editor declared a past co-authorship with one of the authors, MH.
